# Forsythoside A inhibits apoptosis and autophagy induced by infectious bronchitis virus through regulation of the PI3K/Akt/NF-κB pathway

**DOI:** 10.1128/spectrum.01921-23

**Published:** 2023-11-16

**Authors:** Jun Xu, Peng Yin, Xuewei Liu, Xiaolin Hou

**Affiliations:** 1 Department of Veterinary Medicine, Beijing University of Agriculture, Beijing, China; 2 Institute of Microbiology Chinese Academy of Sciences, Beijing, China; University of Georgia, Athens, Georgia, USA

**Keywords:** Infectious bronchitis virus, forsythoside A, apoptosis, autophagy, PI3K/Akt/NF-κB signaling pathway

## Abstract

Infectious bronchitis virus (IBV) causes an acute and highly infectious viral disease of chickens, which brings huge economic losses to the poultry industry. Forsythoside A (FTA) is a natural ingredient with wide pharmacological and biological activities, which has been demonstrated to have anti-IBV activities. In the present study, we found that IBV replication reached the highest level of 7.44 ± 0.11 copies/μL at 48 h post infection (hpi) and caused cytopathic effect and apoptosis in baby hamster kidney (BHK) cells. FTA pretreatment significantly inhibited IBV replication in a dose-dependent manner with an inhibition rate of up to 51.1%. In the presence of interferon deficiency, FTA pretreatment also inhibited IBV replication, indicating that the inhibition of FTA on IBV replication did not rely on the innate immune response of host cells. Additionally, we found that FTA pretreatment attenuated inflammation, apoptosis, and autophagy induced by IBV infection. UV-irradiation-inactivated IBV did not affect the viability of BHK cells, but it upregulated the expressions of phosphoinositide 3-kinase (PI3K), protein kinase B (Akt), and nuclear factor kappa-B (NF-κB) proteins, suggesting that the apoptosis and autophagy induced by IBV infection were through activating the PI3K/Akt/NF-κB pathway. FTA pretreatment downregulated the PI3K/Akt/NF-κB pathway, and the PI3K activator and inhibitor were proven to show that FTA pretreatment inhibited IBV-induced apoptosis and autophagy by attenuating the PI3K/Akt/NF-κB pathway.

**IMPORTANCE:**

Infectious bronchitis virus (IBV) is an acute and highly infectious viral disease that seriously endangered the development of the chicken industry. However, due to the limited effectiveness of commercial vaccines, there is an urgent need to develop safe and effective anti-IBV drugs. Forsythoside A (FTA) is a natural ingredient with wide pharmacological and biological activities, and it has been shown to have antiviral effects against IBV. However, the antiviral mechanism of FTA is still unclear. In this study, we demonstrated that FTA can inhibit cell apoptosis and autophagy induced by IBV infection by regulating the PI3K/AKT/NF-κB signaling pathway. This finding is important for exploring the role and mechanism of FTA in anti-IBV infection, indicating that FTA can be further studied as an anti-IBV drug.

## INTRODUCTION

Avian infectious bronchitis (IB), an acute and highly infectious viral disease, has caused huge economic losses to the worldwide poultry industry since the 1930s (1, 2). Avian infectious bronchitis virus (IBV), the causative agent of IB, is a positive-strand-enveloped RNA virus classified within the gamma coronavirus family. IBV is primarily a respiratory virus, however, some trains can affect the kidney and reproductive tract, causing respiratory distress, kidney damage, and a decrease in egg production (3 – 5). IBV has a high mutation and recombination rate, leading to the generation of new virus strains that are difficult to control. Over 50 serotypes of IBV have been recorded since the first outbreak (6). Currently, although substantial efforts have been undertaken to control and prevent IBV, some commercially available vaccines have shown limited efficacy due to the genetic drift of IBV strains (7). Therefore, the development of effective antivirals against IBV is urgently needed.

To survive and replicate within a host, viruses have evolved various strategies to evade the host’s innate immunity responses (8 – 10). For instance, virus infections can trigger cellular processes such as pyroptosis, necroptosis, apoptosis, and autophagy, which can cause immunosuppression (11 – 14). Studies have reported that the pathogenicity of the IBV is associated with apoptosis and autophagy (9, 15 – 18). IBV is known to restrict apoptosis in infected cells by activating the extracellular signal-regulated kinase (ERK) signaling and upregulating phosphatase dual-specificity phosphatase 6, which induces apoptosis at the late stages of infection (19). Furthermore, IBV triggers the formation of autophagosomes and apparent autophagic flux. The ER stress sensor inositol requiring enzyme 1 is essential for the induction of autophagy (20). Interestingly, the anti-apoptotic ERK1/2 contributes to IBV-induced autophagy (21).

Host cellular kinases can regulate the replication and the relevant pathogenesis of viruses, which can be used to identify the novel therapeutic targets (22 – 25). The phosphatidylinositol-3-kinase and protein kinase B (PI3K/Akt) signaling pathway is one of the most important cellular pathways for viruses and has been shown to participate in various replication steps of multiple viruses. Many viruses have been demonstrated to activate the PI3K/Akt pathway to enhance their entry, uncoating, replication, and persistence in the host (26 – 30). For example, highly pathogenic porcine reproductive and respiratory syndrome virus infection induces biphasic activation of PI3K/Akt in both the early and the late stages, and UV-irradiation-inactivated virus still induces the early event (31). Additionally, the PI3K/Akt pathway also can regulate various cellular processes, including cell proliferation, inflammation, apoptosis, and autophagy (13, 32). Meanwhile, some viruses promote their replication and enhance the apoptotic signaling responses by activating the PI3K/Akt pathway (33). The severe acute respiratory syndrome coronavirus-2 spike has been reported to promote inflammation and apoptosis of host cells through autophagy by suppressing PI3K, Akt, and mammalian target of rapamycin signaling with reactive oxygen species (13). In addition, Newcastle disease virus promotes replication and anti-apoptotic signaling responses by transiently activating of the PI3K/Akt pathway. The replication and infection of IBV are facilitated by activating various host kinases (34 – 36). An article reported that IBV infection is affected by the activation of mitogen-activated extracellular signal-regulated kinase (MEK) and/or PI3K pathway (37). However, the regulation mechanisms between IBV and PI3K/Akt pathway are not fully understood.

Forsythoside A (FTA), the main active ingredient in the traditional Chinese medicine *Forsythia suspense*, exerts various pharmacological activities, including anti-inflammatory, antibacterial, antioxidant, antiviral, and neuroprotective properties through diverse mechanisms (38 – 43). It has been demonstrated to have the antiviral activities against IBV (44, 45). Specifically, FTA can significantly inhibit the replication of IBV *in vivo* and *in vitro* (44, 45). However, the molecular mechanisms underlying its potential anti-IBV effect remain unclear. Moreover, to our knowledge, FTA can suppress proliferation and modulate apoptosis by inhibiting the PI3K/Akt pathway (46, 47). Based on the above, we speculate that FTA regulate cellular processes induced by the replication and infection of IBV by modulating the PI3K/Akt pathway. Our study provides insights into the mechanisms of the anti-IBV effects of FTA and supports its potential therapeutic use in the future. FTA pretreatment can inhibit IBV replication and attenuate the IBV-induced apoptosis and autophagy by inhibiting the PI3K/Akt/NF-κB pathway.

## RESULTS

### IBV infection induced apoptosis of BHK cells through replication

**Fig 1:**
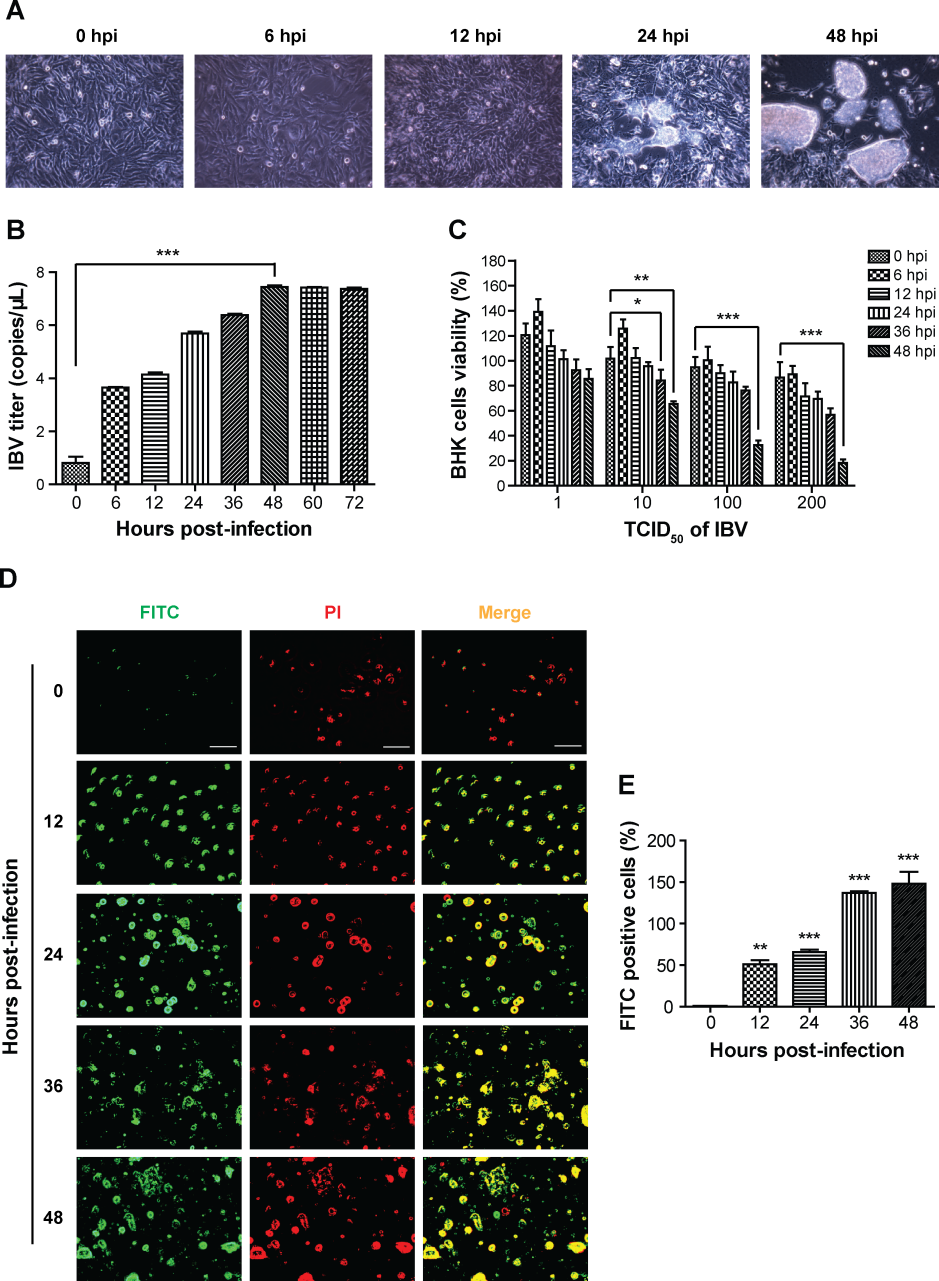
IBV infection and cellular damage in BHK cells. (**A**) IBV infection induced CPE in BHK cells. The BHK cells were infected with 10 TCID_50_ of IBV for 0, 6, 12, 24, and 48 h. The CPE was observed at 12, 24, and 48 hpi, while no CPE was seen at 0 hpi. 200×. (**B**) The copies of IBV in BHK cells were tested by an RT-qPCR assay. The BHK cells were infected with 10 TCID_50_ of IBV for 0, 6, 12, 24, 36, 48, 60, and 72 h. At 48 hpi, the copies reached to the highest level of 7.44 ± 0.11 copies/μL. (**C**) The impact of IBV on BHK cells viability was measured by the MTT assay. BHK cells were infected with IBV (1, 10, 100, and 200 TCID_50_) for different times. IBV infection induced BHK cells death in a time- and dose-dependent manner. At 48 hpi of 10 TCID_50_ IBV, the cell viability was significantly reduced to 65.4%. (**D**) The apoptosis induced by IBV infection was used an immunofluorescence staining assay. BHK cells were infected with 10 TCID_50_ of IBV for 0, 12, 24, 36, and 48 h. Apoptosis cells were labeled with FITC as green, while nuclei were labeled with PI as red. Scale bars: 500 µm. (**E**) The graph represents a quantitative analysis of the number of FITC-positive cells. Data are shown as the mean ± SEM of three independent experiments. *P < 0.05, **P < 0.01, ***P < 0.001 compared with the 0 hpi group.

Firstly, we observed the IBV infection in BHK cells. As shown in Fig. 1A, the CPE was observed at 12 hpi, characterized by a transition of cell morphology from spindle-shaped to round, cell aggregation, detachment, and plaque formation. To study IBV replication in BHK cells, a real-time quantitative polymerase chain reaction (RT-qPCR) assay was used. As shown in Fig. 1B, the copies of IBV were increased with infection time, reaching the maximum of 7.44 ± 0.11 copies/μL at 48 hpi. Next, we established the model of IBV infection by testing the effect of different titers of IBV infection on BHK cells viability at different times. As the results of MTT assay show, IBV infection decreased the viability of BHK cells in a time- and dose-dependent manner (Fig. 1C). In our experiment, the condition for IBV infection was 10 TCID_50_ of IBV infected for 48 h, at this time, the activity of BHK cells decreased to 65.4%. In Fig. 1D and E, the number of FITC-stained positive cells increased with the time of IBV infection. These findings suggested that IBV can infect BHK cells and induce apoptosis through the replication.

### FTA inhibited IBV replication *in vitro*

**Fig 2:**
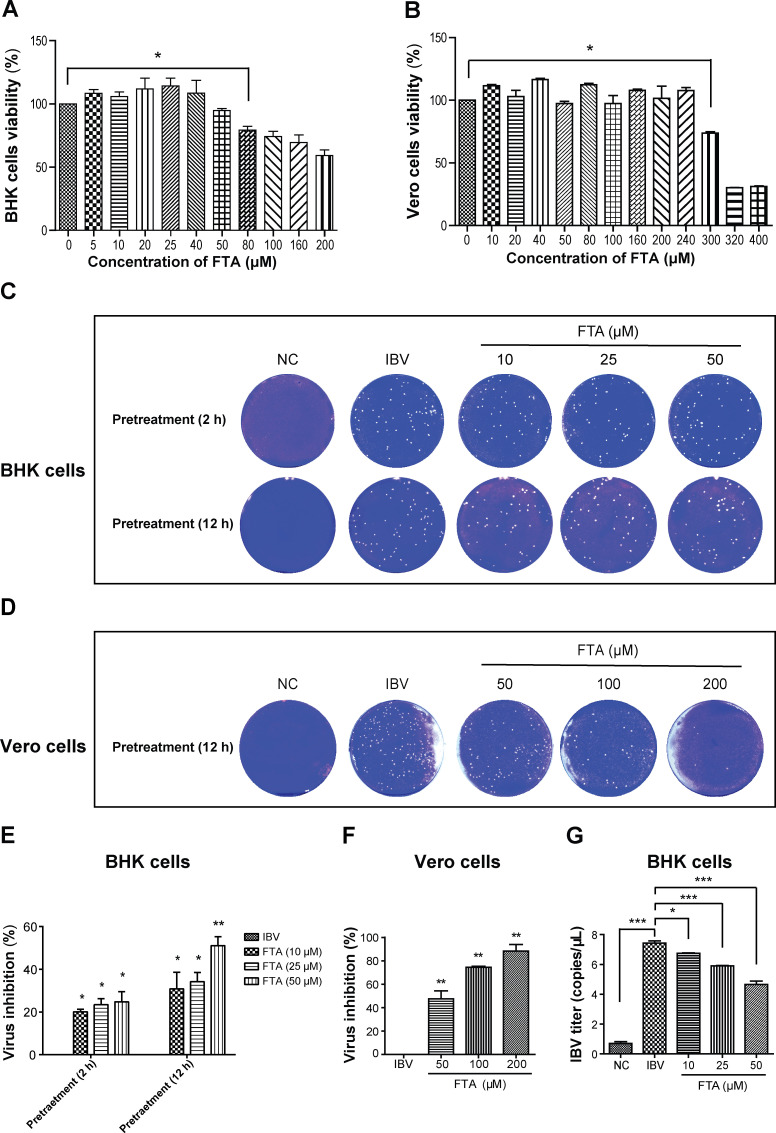
FTA inhibits IBV replication in BHK cells and Vero cells. (**A and B**) The cell viability was measured using the MTT assay. BHK cells and Vero cells were seeded in 96-well plates before treating with various concentrations of FTA for 72 h. The maximum non-cytotoxic concentration of FTA was 50 µM in BHK cells and 240 µM in Vero cells. (**C and D**) The effect of FTA pretreatment on IBV replication was assessed using a plaque assay. BHK cells and Vero cells were treated with FTA for 2 or 12 h before infecting with IBV (10 TCID_50_). After 1 h, the overlay media containing 2% DMEM and 2% methylcellulose were added. Following another 48 h of incubation, the plates were scanned to visualize the plaques. (**E**) The graph represents a quantitative analysis of the virus inhibition percentage of FTA in BHK cells. The replication of IBV was inhibited by FTA pretreatment. After 12 h of FTA pretreatment, the inhibitory rate was significantly up to 51.1%. (**F**) The graph represents a quantitative analysis of the virus inhibition percentage of FTA in Vero cells. FTA pretreatment inhibited IBV replication, and the maximum inhibition rate was 88.4%. (**G**) The graph represents a quantitative analysis of IBV copies in BHK cells. The effect of FTA pretreatment on IBV replication was studied by an RT-qPCR assay. The IBV titers were significantly downregulated in the FTA (10 µM) group. Data are shown as the mean ± SEM of three independent experiments. *P < 0.05, **P < 0.01, ***P < 0.001.

To avoid the cytotoxicity of FTA, an MTT assay was used. Fig. 2A and B showed the maximum concentration of non-cytotoxicity of FTA was 50 µM in BHK cells and 240 µM in Vero cells. Next, we assessed the inhibition of FTA pretreatment on IBV replication in BHK cells. As shown in Fig. 2C and E, FTA pretreatment can inhibit the replication of IBV in BHK cells, with an inhibition rate of 51.1% after 12 h of FTA pretreatment. The RT-qPCR results also showed that FTA can inhibit IBV in a dose-dependent manner (Fig. 2G). Studies have reported that IBV infection is extremely sensitive to the production of cellular interferon (23). To determine whether the inhibition of FTA on IBV is related to the innate immune response of host cells (such as IFN activation), we next discussed the effect of FTA pretreatment on IBV replication in Vero cells, which is an interferon-deficient cell line. As the results show, in Vero cells, FTA pretreatment can also inhibit IBV replication in a dose-dependent manner (Fig. 2D and F). Overall, these results illustrated that the inhibition of FTA on IBV replication does not rely on suppressing IFN production.

### FTA attenuated inflammation, apoptosis, and autophagy induced by IBV infection in BHK cells

**Fig 3:**
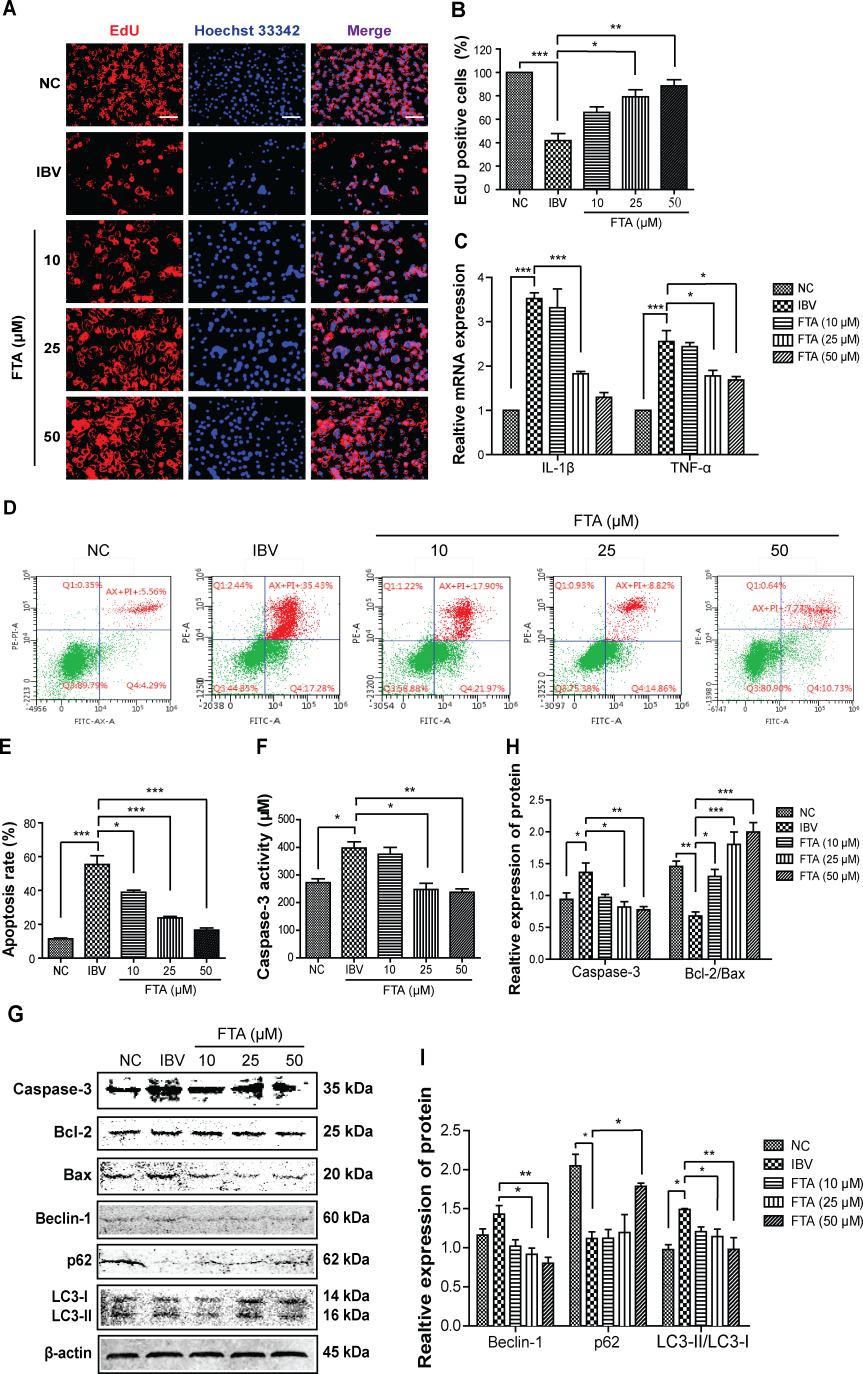
FTA attenuates inflammation, apoptosis, and autophagy induced by IBV infection in BHK cells. The BHK cells were treated with FTA (10, 25, 50 µM) for 12 h and then infected with IBV (10 TCID_50_) for 48 h. (**A**) The effect of FTA on BHK cells proliferation was evaluated using the immunofluorescence staining. The proliferation cell was labeled with EdU as red, while the nucleus was labeled with Hoechst 33342 as blue. Scale bars: 500 µm. (**B**) The graph represents a quantitative analysis of the number of EdU-positive cells. IBV infection significantly inhibited the proliferation of BHK cells, which was notably reversed by FTA pretreatment (25 and 50 µM). (**C**) The mRNA realtive expression level of IL-1β and TNF-α were analyzed via the RT-PCR assay. IL-1β and TNF-α mRNA levels were significantly increased in the IBV group and significantly decreased in the FTA groups with a dose-dependent manner. (**D**) The representative results from FCM. The apoptosis was detected using FITC-labeled Annexin V/PI staining and flow cytometry. (**E**) The graph represents a quantitative analysis of the apoptosis rate. The apoptosis rate was significantly increased to 55.4% in the IBV group, but it was significantly decreased in the FTA groups. (**F**) The graph represents a quantitative analysis of caspase-3 protein. Caspase-3 activity was determined using the Caspase-3 Activity Assay Kit. The caspase-3 level was significantly increased in the IBV group, and it was significantly decreased in the FTA groups in a dose-dependent manner. (**G**) The level of BAX, Bcl-2, caspase-3, Beclin-1, p62, and LC3B proteins in BHK cells were analyzed by western blotting. (**H and I**) The graph presents a quantitative analysis of the expression of indicated proteins. Data are expressed as mean ± SEM from three independent experiments. *P < 0.05, **P ˂ 0.01, ***P < 0.001.

The EdU immunofluorescence stain assay was used to study the effect of FTA pretreatment on cell proliferation. As shown in Fig. 3A and B, compared with the NC group, the proliferation of BHK cells was significantly downregulated in the IBV group (*P* < 0.001), but it was notably reversed in FTA (25 and 50 µM) groups (*P* < 0.05). The mRNA expression of pro-inflammatory cytokines were assayed by a real-time polymerase chain reaction (RT-PCR) assay. In Fig. 3C, IBV infection significantly upregulated the level of IL-1β and TNF-α mRNA (*P* < 0.001), but FTA pretreatment can downregulate them in a dose-dependent manner (*P* < 0.01).

Additionally, to study the effect of FTA on apoptosis induced by IBV infection, we analyzed the apoptosis rate using the FCM assay, and detected the level of pro-apoptotic proteins (caspase-3 and BAX) and anti-apoptotic protein (Bcl-2) using the western blot assay and caspase-3 activity assay kit. In Fig. 3D and E, IBV infection upregulated the apoptosis rate to 55.4% (*P* < 0.001), while FTA pretreatment downregulated it in a dose-dependent manner (*P* < 0.05). In Fig. 3F through H, compared with the NC group, the level of caspase-3 protein was increased and Bcl-2/BAX protein was decreased in the IBV infection group (*P* < 0.0.5), while FTA pretreatment can significantly block the trends (*P* < 0.05).

To further investigate the effect of FTA pretreatment on autophagy induced by IBV, we tested the levels of Beclin-1, p62, and LC3B proteins using the western blot assay. As shown in Fig. 3, compared with the NC group, the relative expression of Beclin-1 and LC3-II/LC3-I proteins showed a significant upregulation (*P* < 0.05), and p62 protein was significantly downregulated in the IBV group (*P* < 0.05). However, FTA pretreatment was ameliorated the trends (*P* < 0.05).

In conclusion, these data showed that FTA pretreatment can attenuate the inflammation, apoptosis, and autophagy induced by IBV infection in BHK cells.

### FTA inhibited the activation of PI3K/Akt/NF-κB signal pathway induced by IBV infection

**Fig 4:**
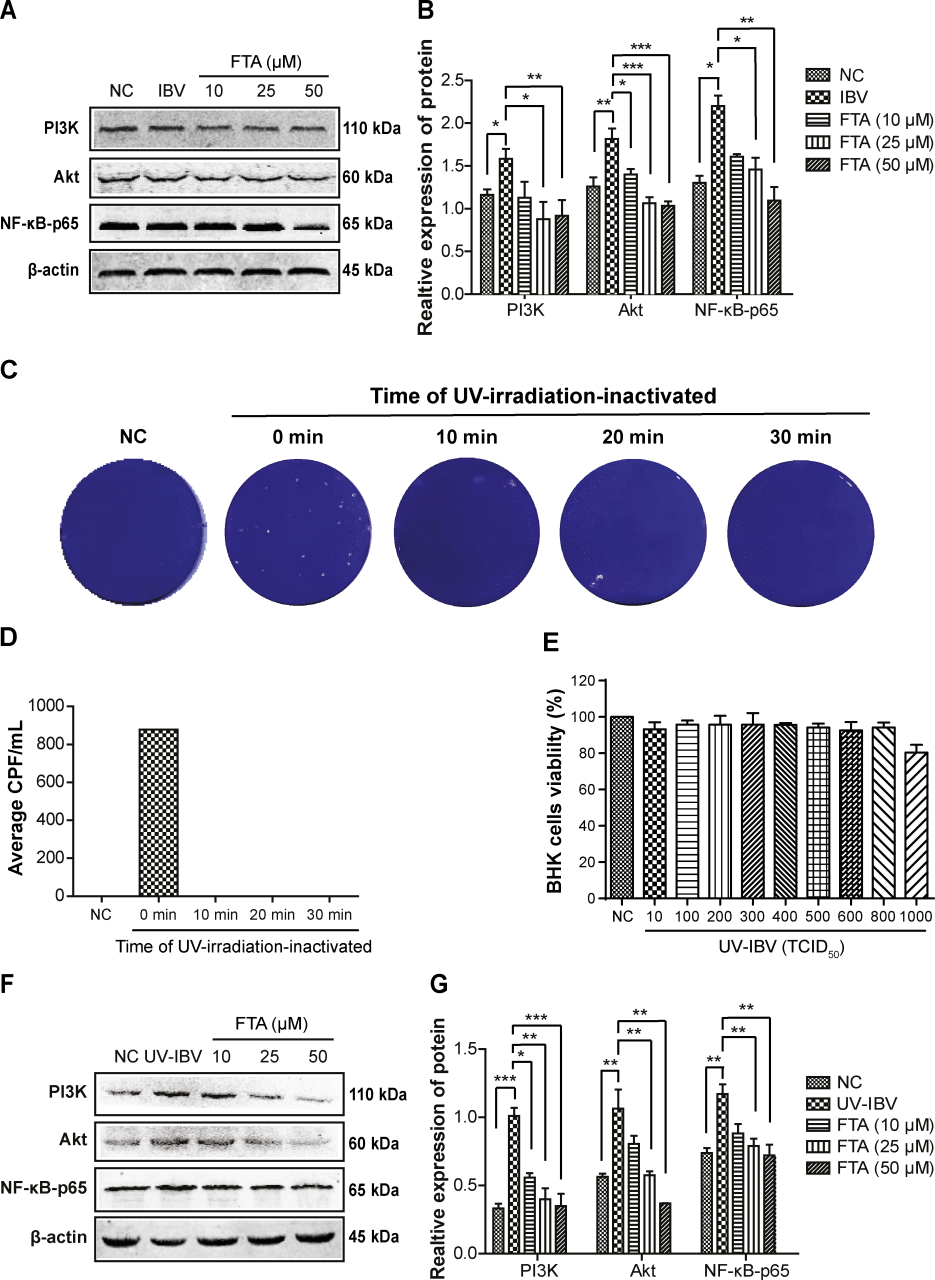
FTA downregulates the over-expression of the PI3K/Akt/NF-κB-p65 pathway induced by IBV infection in BHK cells. The BHK cells were treated with FTA (10, 25, 50 µM) for 12 h and then infected with 10 TCID_50_ of IBV or UV-irradiation-inactivated IBV for 48 h. (**A**) The level of PI3K, Akt, and NF-κB-p65 proteins were analyzed by western blotting assay. The β-actin image was reused because it is part of the internal control experiment in Fig. 3G. (**B**) The graph represents a quantitative analysis of the indicated protein expression levels. PI3K, Akt, and NF-κB-p65 levels were significantly increased in the IBV-infected group and significantly decreased at FTA groups in a dose-dependent manner. (**C**) The infection time of UV-irradiation-inactivated virus was determined using a plaque test. The IBV (10 TCID_50_) was exposed to UV radiation for 0, 10, 20, and 30 min, and all plates were scanned to visualize the plaques. (**D**) The graph represents a quantitative analysis of the average CPE in UV-irradiation-inactivated IBV-infected cells. IBV exposure to UV radiation for 10 min did not cause CPE. (**E**) The cell viability was measured using an MTT assay. The viability of BHK cells was not affected by different TCID_50_ of UV-irradiation-inactivated IBV. (**F**) The expressions of PI3K, Akt, and NF-κB-p65 proteins were analyzed by western blotting assay. (**G**) The graph represents a quantitative analysis of the indicated protein expression levels. PI3K, Akt, and NF-κB-p65 levels were significantly increased in UV-irradiation-inactivated IBV-infected cells and significantly decreased in FTA treatment groups with a dose-dependent manner. Data are shown as the mean ± SEM of three independent experiments. *P < 0.05, **P ˂ 0.01, ***P < 0.001.

To investigate the mechanism of FTA protecting BHK cells from IBV infection, we focused on the expression of PI3K/AKT signaling pathway. In Fig. 4A and B, compared to the NC group, the level of PI3K, AKT, and NF-κB-p65 proteins were significantly upregulated in the IBV group (*P* < 0.05) while downregulated in a dose-dependent manner in the FTA groups (*P* < 0.05).

To further determine the relationship between FTA’s inhibition and PI3K/Akt/NF-κB, UV-irradiation-inactivated IBV was used to infect BHK cells. We found that UV-irradiation-inactivated IBV did not affect the BHK cell viability (Fig. 4C, D and E), but it significantly upregulated the levels of PI3K, Akt, and NF-κB-p65 proteins (*P* < 0.01) (Fig. 4F and G). And FTA pretreatment also significantly blocked these trends (*P* < 0.05).

In conclusion, all results indicate that the PI3K/Akt/NF-κB signal pathway was activated by IBV infection, and FTA can inhibit it.

### FTA inhibited apoptosis and autophagy induced by IBV infection via attenuating the PI3K/Akt/NF-κB signaling pathway

**Fig 5:**
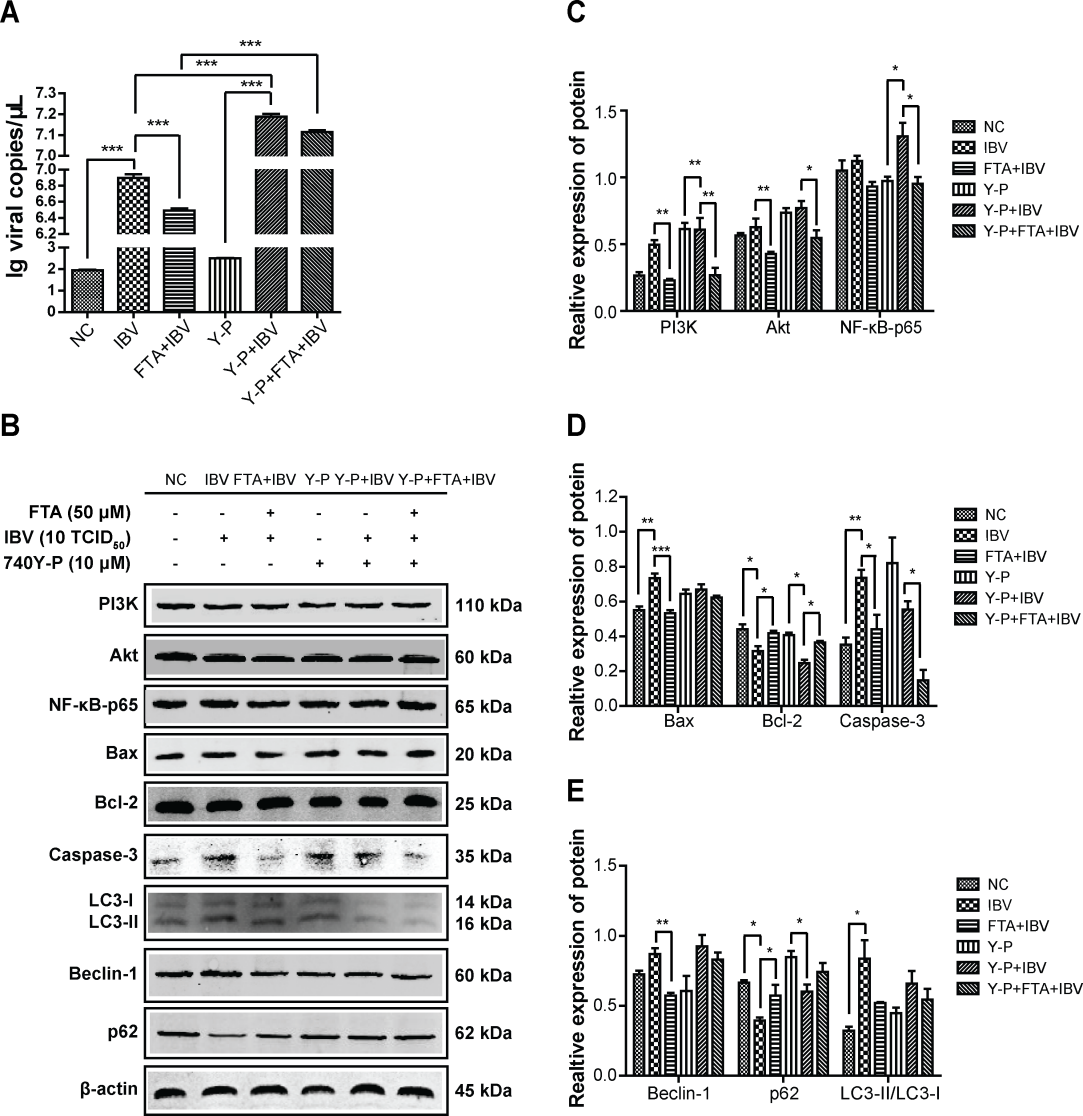
The PI3K activator 740Y-P obviously impacted on the apoptosis and autophagy-related proteins induced by IBV infection in BHK cells. The BHK cells were treated with 740Y-P (10 µM) for 1 h, and/or treated with FTA (50 µM) for 12 h before infecting with IBV (10 TCID_50_) for 48 h. (**A**) The level of IBV-N mRNA was detected by RT-PCR assay. The expression of IBV-N mRNA was significantly inhibited by FTA (50 µM), whlie 740Y-P (10 µM) activated it. (**B**) The expression level of PI3K, Akt, NF-κB-p65, caspase-3, BAX, bcl-2, p62, Beclin-1, and LC3B proteins were analyzed by western blotting assay. (**C–E**) The graph represents a quantitative analysis of the band intensity. Data are shown as the mean ± SEM of three independent experiments. *P < 0.05, **P ˂ 0.01, ***P < 0.001.

**Fig 6:**
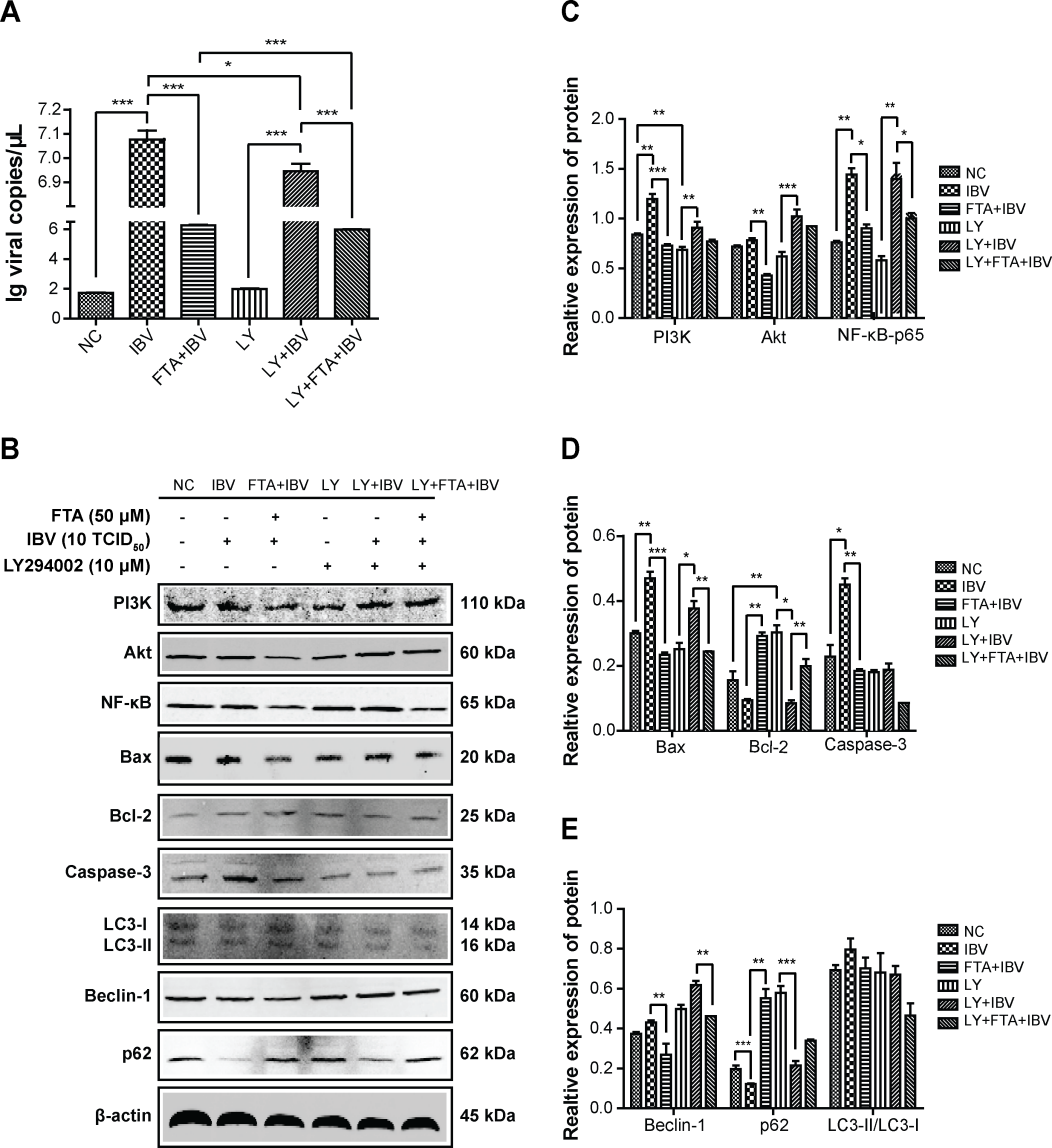
The PI3K inhibitor LY294002 obviously affected the apoptosis and autophagy-related proteins induced by IBV infection in BHK cells. The BHK cells were pretreated with LY294002 (10 µM) for 2 h, and/or treated with FTA (50 µM) for 12 h before infecting with IBV (10 TCID_50_) for 48 h. (**A**) The level of IBV-N mRNA was detected using RT-PCR assay. The expression of IBV-N mRNA was significantly inhibited by FTA (50 µM) and LY294002 (10 µM). (**B**) The expression levels of PI3K, Akt, NF-κB-p65, caspase-3, BAX, bcl-2, p62, Beclin-1, and LC3B proteins were analyzed by western blotting assay. (**C–E**) The graph represents a quantitative analysis of the band intensity. Data are shown as the mean ± SEM of three independent experiments. *P < 0.05, **P ˂ 0.01, ***P < 0.001.

To determine the relationship between the mechanism of FTA inhibiting apoptosis and autophagy induced by IBV and the PI3K/AKT/NF-κB pathway, the PI3K activator 740Y-P (10 µM) and PI3K inhibitor LY294002 (10 µM) were used to over-express or inhibit expression of PI3K protein. In Fig. 5A and 6 A, the PI3K agonist 740Y-P increased the expression of IBV-N mRNA, while the PI3K inhibitor LY294002 decreased it, similar to FTA pretreatment. These findings indicated that FTA inhibited IBV replication by inhibiting the PI3K protein.

Additionally, the PI3K activator 740Y-P exacerbated the expressions of PI3K, Akt, and NF-κB-p65 proteins induced by IBV infection. It also enhanced the apoptosis and autophagy of BHK cells. However, these changes were attenuated by FTA pretreatment (Fig. 5B through E). FTA and 740Y-P had opposite effects on IBV infection. On the contrary, the PI3K inhibitor LY294002 suppressed the expression of PI3K, Akt, and NF-κB-p65 proteins induced by IBV infection. It also reduced the apoptosis and autophagy of BHK cells, similar to FTA (Fig. 6B through E). Since, the changes in Akt and NF-κB had the same kinetics pattern as PI3K, PI3K is the upstream inducer of Akt/NF-κB.

In summary, our results findings indicated that IBV infection induced autophagy and apoptosis partially through the PI3K/Akt/NF-κB signaling pathway, while FTA pretreatment prevented these effects.

## DISCUSSION

IBV brings huge economic losses to the poultry industry. However, the commercially available vaccines have shown limited efficacy. Although the antiviral activities against IBV of FTA have been demonstrated, the diverse mechanisms are still unclear. Our study aims to explore the effect and mechanism of FTA on IBV infection in the BHK cells. We found that IBV could infect BHK cells and induce apoptosis at 12 hpi. Additionally, FTA pretreatment for 2 and 12 h significantly inhibited IBV replication. Further research proved that this was not dependent on the innate immune response of host cells, such as IFN activation. Furthermore, our experiments demonstrated that FTA can significantly inhibit cellular processes induced by IBV infection such as inflammation, apoptosis, and autophagy. These effects may be achieved by regulating the PI3K/Akt/NF-κB signaling pathway. Overall, these results were consistent with previous studies (44, 45), indicating the potential of FTA as an anti-IBV drug for further research.

Autophagic is a complex and evolutionarily conserved cellular pathway that culminates in the lysosomal degradation of selected substrates. In general, the autophagy pathway can be divided into five distinct stages, including induction, autophagosome formation, cargo selection, lysosomal fusion, and degradation (48). LC3 is a broadly used marker for autophagosome detection. It is cleaved, lapidated, and incorporated into maturing autophagosomes (48). Beclin-1 promotes autophagosome formation, while p62 is a substrate of autophagy and continues to degrade (49). Coronaviruses have been shown to interact with the cellular autophagy pathway to promote their replication (50). However, if the dysfunctional formation of autophagosomes exceeds the lysosome’s degradation capacity, apoptosis may occur. Previous studies have reported that IBV can replicate and induce apoptosis *in vitro* (34, 51). In this study, we found that IBV infection significantly impaired the viability and proliferation of BHK cells, accompanied by excessive autophagy and apoptosis, indicating disruption of cellular homeostasis. However, FTA pretreatment can reverse this damage. Therefore, we confirm that FTA can protect BHK cells from the damage induced by IBV infection.

The PI3K/Akt pathway plays a two-faced role in the interactions between virus and cells (52). On one hand, it promotes virus replication, while on the other hand, it can inhibit virus replication as an active part of the immune response. In our experiment, the PI3K/Akt pathway was activated when BHK cells were infected with IBV or UV-irradiation-inactivated IBV. Furthermore, the PI3K activator promoted IBV replication, while PI3K inhibitor can reduce IBV replication. Therefore, we suggested that the PI3K/Akt pathway may facilitate the entry and replication of IBV by regulating the endocytosis of BHK cells. However, FTA pretreatment for 2 and 12 h significantly inhibited IBV replication and the activity of PI3K/Akt pathway. If FTA directly inactivated IBV, it would be unlikely that FTA could reverse the activated PI3K/Akt signaling pathway. Therefore, we speculate that the inhibitory effect of FTA on IBV replication is due to its inhibition of the PI3K/Akt pathway. Furthermore, to confirm this, we used the UV-irradiation-inactivated IBV to infect cells.

Meanwhile, growing evidence has shown that the PI3K/Akt pathway can stimulate the production of inflammatory cytokines by contributing to the activation of the NF-kB transcription factor (53). For instance, avian reovirus activates Akt to increase the production of proinflammatory cytokines IL-6 and IL-1β (54). In this study, we found that the expression of NF-κB, IL-1β, and TNF-α was upregulated during IBV infected, and FTA was reversed it. These findings suggest that IBV infection induces a pro-inflammatory response by triggering the PI3K/Akt/NF-κB signal pathway. Although the exact receptor modulated by FTA has not been defined, facts have proved that FTA regulation of the PI3K/Akt/NF-κB signal pathway is effective.

In conclusion, IBV induces PI3K/Akt/NF-κB signal transduction activation to promote its infection, leading to apoptosis and autophagy. FTA restricts IBV infection by inhibiting PI3K/Akt/NF-κB signaling pathway, thereby attenuating IBV-induced apoptosis and autophagy. Additionally, our experiment had certain limitations because choosing the BHK cells as the host cells for IBV, which did not fully represent all reactions of IBV infection. Although reports have demonstrated that FTA has an anti-IBV effect by inhibiting IBV replication, further research is still needed to explore FTA as a potential anti-IBV drug.

## MATERIALS AND METHODS

### Reagents

The FTA was purchased from Chengdu Desite Biotechnology Co., Ltd (Sichuan, China) and was obtained highly purified (≥98%) with an average molecular weight of 624.59. The PI3K agonist 740Y-P (7) was purchased from Selleck Chemicals (Houston, Texas, USA) with a high level of purify (98.38%) and an average molecular weight of 3270.7. The PI3K inhibitor LY294002 (21) was purchased from Sigma-Aldrich (St. Louis, MO, USA), respectively. It was also obtained highly purified (≥98%) with an average molecular weight of 307.34.

The 3-(4, 5-dimethylthiazol-2-yl)−2, 5-diphenyl-tetrazolium bromide (MTT) was purchased from Sigma-Aldrich (St. Louis, MO, USA). The pMD18-T plasmids expressing IBV-N were purchased by Sanhon Biotech (Beijing, China).

### Cells and virus lines

A fibroblast cell line baby hamster kidney (BHK) cells (Cat No. FS-0185) and the African green monkey kidney (Vero) cells (Cat No. CCL-81) were purchased from American Type Culture Collection (ATCC, Manassas, VA, USA). The cells were cultured in Dulbecco’s modified Eagle medium (DMEM; Thermo Fisher Scientific, USA), supplemented with 10% fetal calf serum (FBS; Thermo Fisher Scientific, USA) and 1% penicillin-streptomycin (PS; Thermo Fisher Scientific, USA) in a constant temperature incubator at 37°C with a humidified atmosphere containing 5% CO_2_.

The IBV strain Beaudette US (Cat No. VR-22) was obtained from the ATCC (11, 24), amplified and titrated in BHK and Vero cells. BHK cells were cultured in DMEM which was supplemented with 10% FBS and 1% PS for 24 h and then infected with 10 TCID_50_ of IBV and periodic rocking. After incubation for 1 h, the cells were washed thrice with phosphate buffer saline (PBS; Hyclone, China) to remove any unbound virus and replenished with fresh DMEM supplemented with 3% FBS. Subsequently, the cytopathic effect was observed under a light microscope (Thermo Fisher Scientific, USA).

### Cell treatment

**Fig 7:**
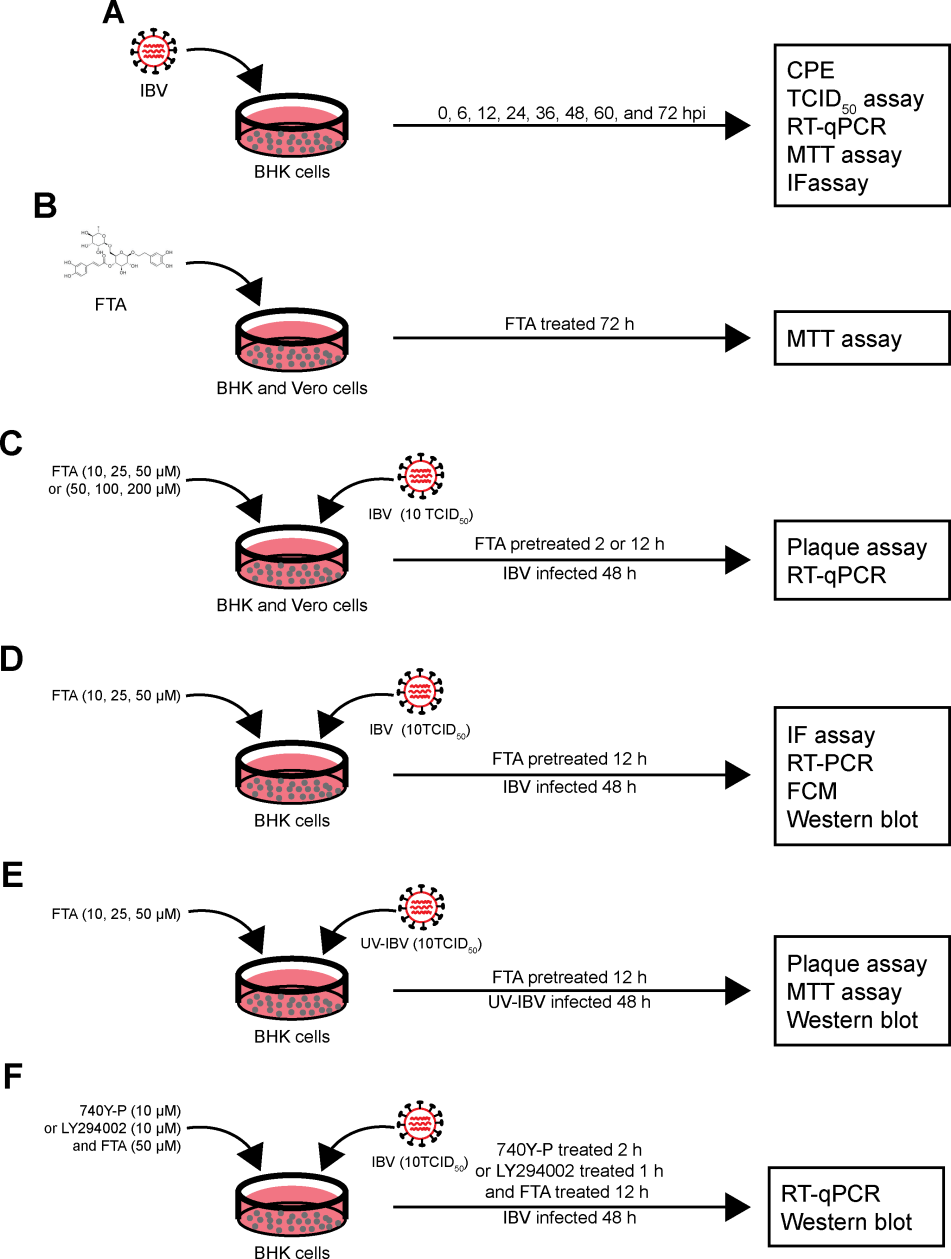
Schematic presentation of the experimental design. (**A**) BHK cells were infected with IBV for 0, 6, 12, 24, 36, 48, 60, and 72 h to determine the titer and time of IBV infection. (**B**) BHK cells and Vero cells were treated with FTA for 72 h to determine the non-cytotoxicity concentration. (**C**) BHK cells and Vero cells were treated with FTA for 2 or 12 h before infecting with IBV (10 TCID_50_) for 48 h to determine the inhibition of FTA on IBV replication. (**D**) BHK cells were treated with FTA (10, 25, and 50 µM) for 12 h before infecting with IBV (10 TCID_50_) for 48 h to determine the effect of FTA on apoptosis and autophagy induced by IBV infection. (**E**) BHK cells were treated with FTA (10, 25, and 50 µM) for 12 h before infecting with UV-IBV (10 TCID_50_) for 48 h to determine the effect of FTA on PI3K/Akt pathway activated by IBV infection. (**F**) BHK cells were treated with 740Y-P (10 µM) for 1 h or LY294002 (10 µM) for 2 h and treated with FTA (50 µM) for 12 h before infecting with IBV (10 TCID_50_) for 48 h to determine the mechanism of FTA on IBV infection.

The design of this experimental as shown in Fig. 7. To determine the titer and time of IBV infection, BHK cells were infected with IBV (1, 10, 100, and 200 TCID_50_) for 0, 6, 12, 24, 36, 48, 60, and 72 h (Fig. 7A). To determine the non-cytotoxic concentration of FTA, BHK cells were treated with FTA (0, 5, 10, 20, 25, 40, 50, 80, 100, 160, and 200 µM) for 72 h, and Vero cells were treated with FTA (0, 10, 20, 40, 50, 80, 100, 160, 200, 240, 300, 320, and 400 µM) for 72 h (Fig. 7B).

In FTA anti-IBV research, the experiment divided into three groups: negative control group (NC), positive control group of IBV or UV irradiation inactivated IBV infection (IBV or UV-IBV), and FTA treatment for IBV infection groups (FTA). In the NC group, cells were not subjected to any treatment. In IBV or UV-IBV group, cells were infected with 10 TCID_50_ of IBV or UV-IBV for 48 h. In FTA groups, cells were treated with FTA (10, 25, and 50 µM in BHK cells; 50, 100, and 200 µM in Vero cells) for 2 or 12 h prior IBV infection (Fig. 7C to E).

In FTA anti-IBV mechanism research, the experiment divided into six groups: negative control group (NC), positive control group of IBV infection (IBV), FTA treatment for IBV infection group (FTA + IBV), PI3K activator 740Y-P or PI3K inhibitor LY294002 treatment group (Y-P or LY), FTA PI3K activator or PI3K inhibitor treatment for IBV infection group (Y-P + IBV or LY + IBV), and combination of PI3K activator or PI3K inhibitor and FTA treatment of IBV infection group (Y-P + FTA + IBV or LY + FTA + IBV). In the NC group, BHK cells were not subjected to any treatment. In the IBV group, BHK cells were infected with 10 TCID_50_ of IBV for 48 h. In FTA + IBV group, BHK cells were treated with FTA (50 µM) for 12 h before IBV infection. In Y-P or LY group, BHK cells were treated with PI3K activator 740Y-P (10 µM) for 2 h or PI3K inhibitor LY294002 (10 µM) for 1 h. In Y-P + IBV or LY + IBV group, BHK cells were treated with PI3K activator or PI3K inhibitor before IBV infection. In Y-P + IBV + FTA or LY + IBV + FTA group, BHK cells were treated with PI3K activator or PI3K inhibitor before FTA treatment and then infected with IBV (Fig. 7E).

### The 50% tissue culture infectious dose (TCID_50_) assay

BHK cells and Vero cells were seeded in 96-well plates and infected with serial 10-fold dilutions of samples in 8 replicates. The plates were incubated for approximately 7 days at 37°C, and the titers of IBV were calculated using the Reed-Muench method and are shown as the TCID_50_/mL.

### Cytotoxicity analysis

The cytotoxicity of IBV, FTA, and UV-irradiation-inactivated IBV on BHK or Vero cells was evaluated using the MTT assay (34). Briefly, the cells were seed in 96-well plates at a density of 2 × 10^4^ CFU/well and treated with IBV, UV-irradiation-inactivated IBV, or FTA for 0, 6, 12, 24, 36, 48, 72 h, respectively. Subsequently, the cells were incubated with colorless DMEM (80 µL/well) and 5 mg/mL MTT (20 µL/well) solution at 37°C for 4 h. After washing three times with PBS, 0.05% DMSO (100 µL/well) was added, and the plate was gently shaken for 10 min. The optical density (OD) value of wells at 490 nm was measured using an enzyme-linked immunosorbent assay reader (Thermo Fisher Scientific, USA) (31). The percentage of viable cells was calculated according to the equation: the percentage of viable cells = (OD value of experiment groups/OD value of negative control group) × 100% (9).

### Plaque assay

The replication of IBV in BHK and Vero cells was tested by the plaque assay. IBV-infected cells were seeded in 6-well plates and overlaid with DMEM containing 3% FBS and 3% methyl cellulose (17). The plates were incubated at 37°C for 72 h, after which the plaque-forming units (PFU) were counted. Cells were stained with 1% crystal violet for 1 h before counting. The virus inhibition percentage was calculated as follows: [1 − (PFU of experiment group)/(PFU of negative control group)] × 100% (23).

### Immunofluorescence (IF) assay

The IF assay was performed to determine the apoptosis and proliferation of BHK cells. Cell apoptosis was tested using commercial Annexin V-FITC/propidium iodide (AV/PI) dual staining commercial kits (Beyotime, Beijing, China). BHK cells were seeded in 6-well plates and infected with 10 TCID_50_ of IBV for 0, 12, 24, 36, and 48 h. Following the manufacturer’s instructions, the BHK cells were digested with trypsin, collected by centrifugation, and washed with PBS. Then stained with annexin V-FITC and PI, all cells were visualized by a fluorescence microscope.

Cell proliferation was assayed using the BeyoClick EdU-488 Cell Proliferation Assay Kit (Beyotime) (55). The BHK cells were pretreated with FTA (10, 25, and 50 µM) for 12 h before infecting with 10 TCID_50_ of IBV for 48 h. Following the user manual, all cells were labeled by EdU and Hoechst 33342 and observed under fluorescence microscope.

All results were visualized by a fluorescence microscope (Olympus Corporation, Japan) at a magnification of 400×, and the signals were counted in five random visional fields. The Image pro software (USA) was used to analyze these images.

### Caspase-3 activity analysis

The activity of caspase-3 protein in BHK cells was detected using Caspase-3 Assay Kit (Beyotime Biotechnology, China). Briefly, after the BHK cells in 96-well plates were treated with FTA (10, 25, and 50 µM) and IBV (10 TCID_50_), the 50 µL of cellular lysate supernatant, 40 µL of detection buffer, and 10 µL of caspase-3 substrate acetyl-Asp-Glu-Val-Asp p-nitroanilide (2 mM) were added into the plates and incubated for 60 min (40). The absorbance of *p*-nitroanilide was determined at 405 nm using a microtiter plate reader. The caspase-3 activity was calculated as a ratio of *p*-nitroanilide content to total protein amount (44).

### Flow cytometry assay

The flow cytometry (FCM) assay was used to test the apoptosis of BHK cells. The Annexin V-FITC/propidium iodide (AV/PI) dual-staining commercial kits were purchased from Beyotime Biotechnology (26). Following the manufacturers’ instructions, briefly, after treatment with FTA (10, 25, and 50 µM) and IBV (10 TCID_50_), the BHK cells were digested with trypsin, collected by centrifugation, and washed with PBS. Then stained with annexin V-FITC and PI, the cells were analyzed by FCM (Becton Dickson). The cells were divided into (annexin V/PI) early (annexin V positive and PI-negative) and late apoptotic (annexin V positive and PI-positive).

### Real-time polymerase chain reaction assay

To determine the expression level of IBV-N, IL-1β, and TNF-α mRNA, 1 × 10^6^ cells were seeded in 6-well plates for at least 12 h before treatment with IBV (10 TCID_50_) and FTA (10, 25, and 50 µM). Total RNA from cells was isolated using the RNApure Tissue & Cell Kit (ComWin Biotech, Beijing, China) and SteadyPure Virus DNA/RNA Extraction Kit (Accurate Biology, Beijing, China). Additionally, total RNA was used for reverse transcription using HiFi-MMLV cDNA Kit (ComWin Biotech, Beijing, China) and oligo-dT priming as per the manufactures’ instructions. For qPCR, cDNA was amplified using UltraSYBR Mixture (ComWin Biotech, Beijing, China) in Bio-Rad IQ5 Thermal Cycler (Bio-Rad) (5, 27). The primers were listed in Table 1.

**TABLE 1:** Sequences of chicken primer pairs used for real-time polymerase chain reaction (RT-PCR)

Gene	Forward primer (5'−3')	Reverse primer (5'−3')	Bp
IL-1β	GCAGGCAGTATCACTCATTGT	GGCTTTTTTGTTGTTCATCTC	114
TNF-α	GCCTCTTCTCATTCCTGCTTGTGG	GTGGTTTGTGAGTGTGAGGGTCTG	149
IBV-N	GAACAGGACCAGCCGCTAA	GAGGAATGAAATCCCAACG	189
β-Actin	CCCAAAGCCAACAGAGAGAA	CCATCACCAGAGTCCATCAC	140

The β-actin was used as a reference gene. The expression fold changes were calculated using the 2^−∆∆*CT*^ method or *y* = −3.3049*x* + 36.652 (*x* = lg viruses/μL, *y* = CT).

### Western blotting

The total protein lysate from BHK cells was extracted using an extraction kit (Nanjing Key Gen Biotech, Nanjing, China) and quantified by using a BCA protein assay kit (Aid lab Biotechnologies, Beijing, China). The β-actin images were reused because they are part of the same internally controlled experiment. The protein samples (20 µg/lane) were separated on 8%–15% polyacrylamide gel electrophoresis (Applygen Technologies, Beijing, China) and transferred onto nitrocellulose filter membranes (NC; Pierce Biotechnology, Inc., USA). After that, NC membranes were incubated with primary antibodies overnight at 4°C and a secondary antibody at room temperature for 40 min (45). All images of the membranes were displayed by the Odyssey dual color infrared fluorescence imaging system (LICOR, USA) and analyze using the ImageJ software (Bethesda, USA).

The primary antibodies against the following proteins were used: rabbit anti-β-actin (1:1,000; Cell Signaling Technology [CST], Boston, Mass, USA), rabbit anti-caspase-3 (1:1,000; CST), rabbit anti-Bcl-2 (1:1,000; CST), rabbit anti-BAX (1:1,000; CST), rabbit anti-Beclin-1 (1:1,000; CST), rabbit anti-p62 (1:1,000; CST), rabbit anti-LC3 (1:1,000; CST), rabbit anti-PI3K (1:1,000; CST), rabbit anti-Akt antibod (1:1,000; CST), and rabbit anti-NF-κB-p65 (1:1,000; CST). The secondary antibodies were used: Alexa Fluor488 labeled goat anti-rabbit IgG (H + L) (1:1,000; Life Technologie, Waltham, Mass, USA).

### Statistical analysis

All data are presented as the mean ± standard error of the mean (SEM) from three independent experiments performed. Statistical calculations were performed using GraphPad Prism version 8 (San Diego, CA, USA) (43). Student’s *t*-test was used for comparisons of two sample means. One-way ANOVA analysis with Dunnett’s test was used for multiple comparison. *P*-values <0.05 was considered statistically significant. The samples were randomly divided into different groups by the random number method. Investigators were not blinded during the experiment and outcome assessment. Most experiments were repeated with similar results.

## Data Availability

The data used to support the findings of the current study are available from the corresponding author on reasonable request.
